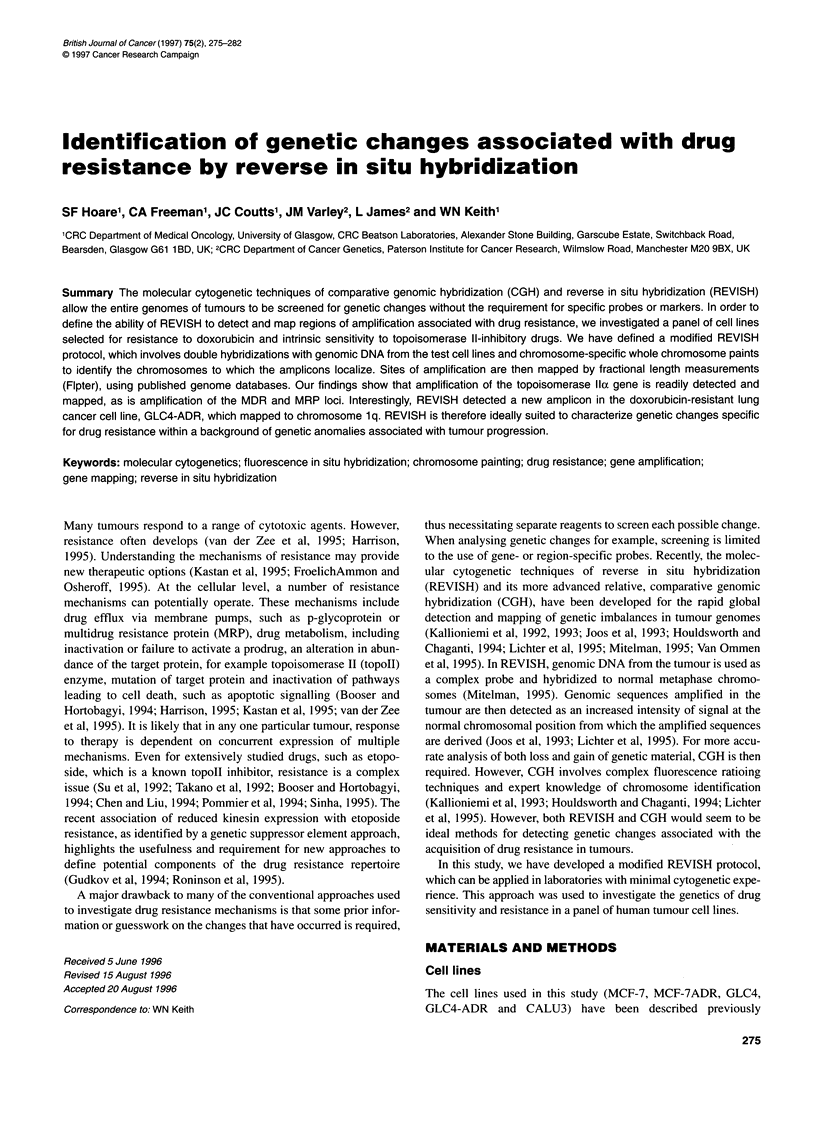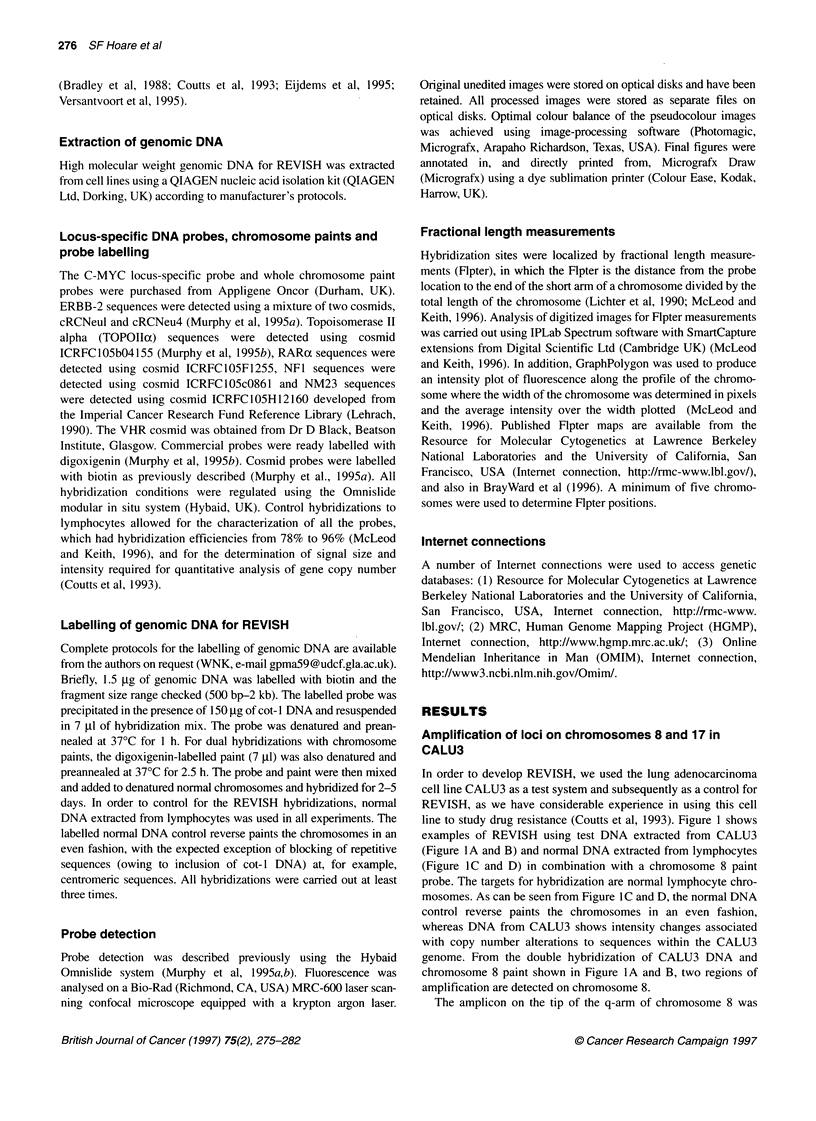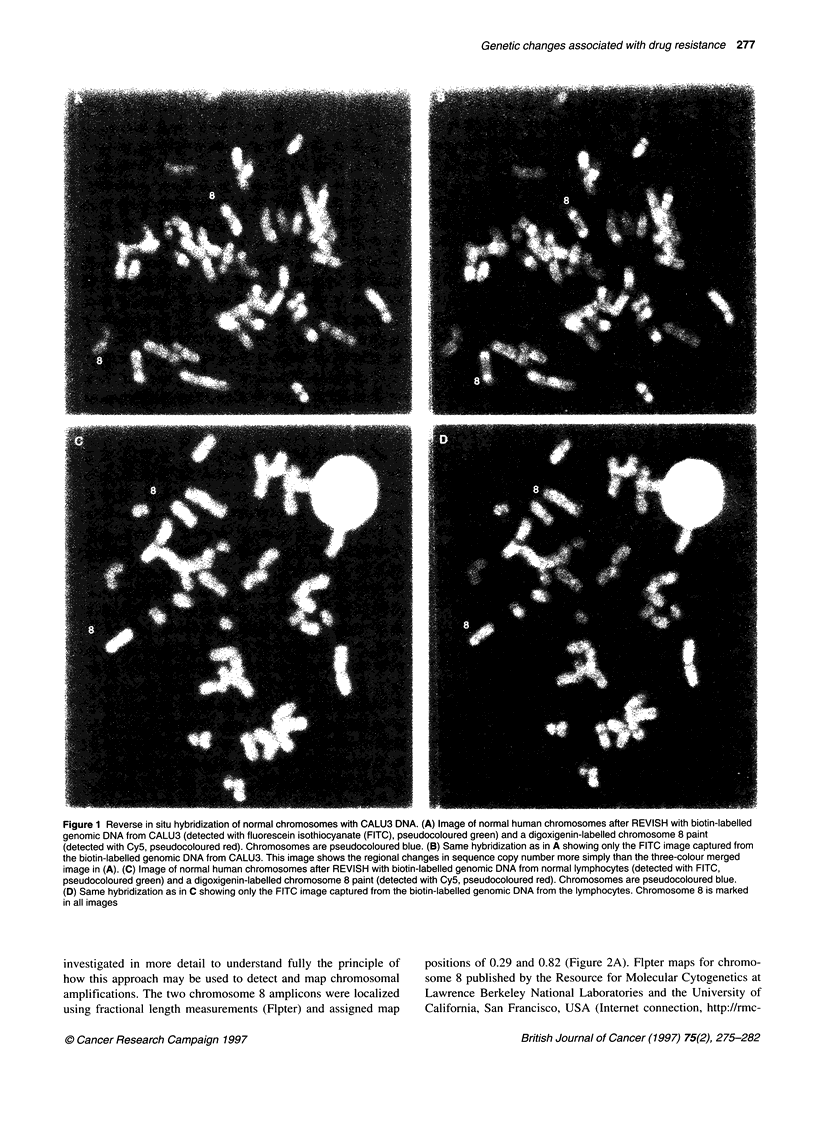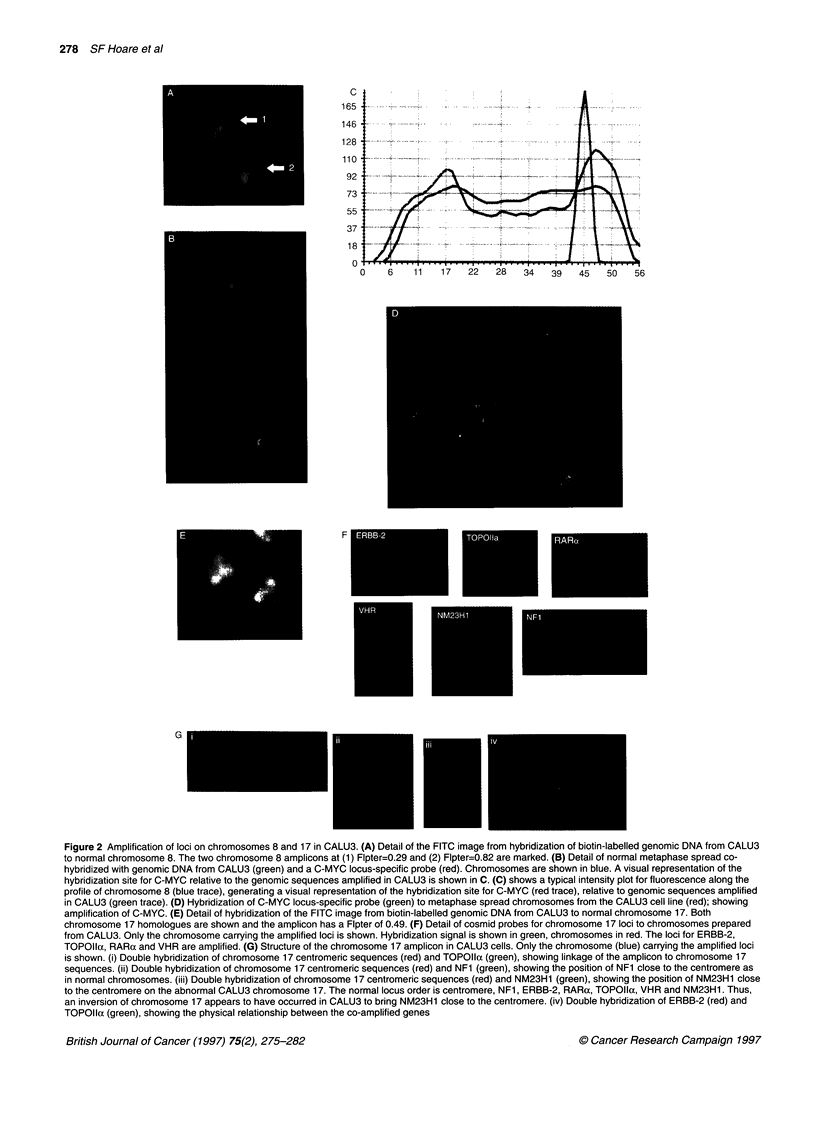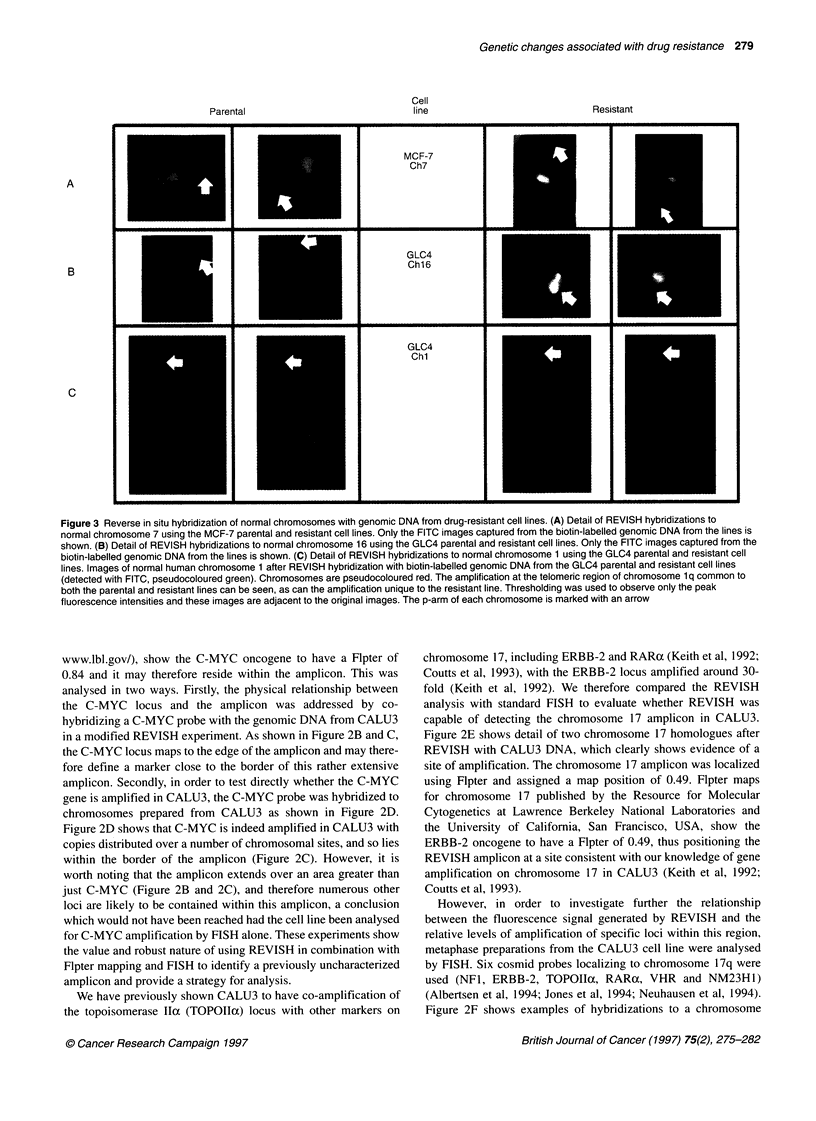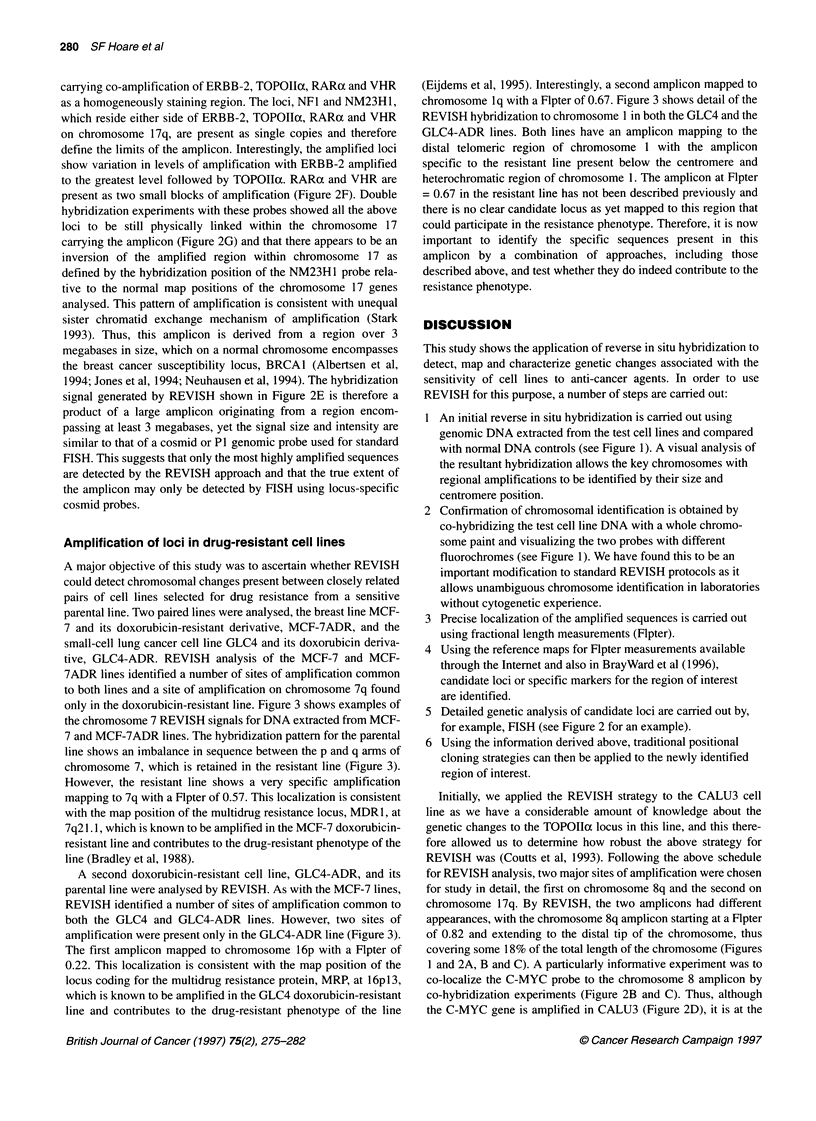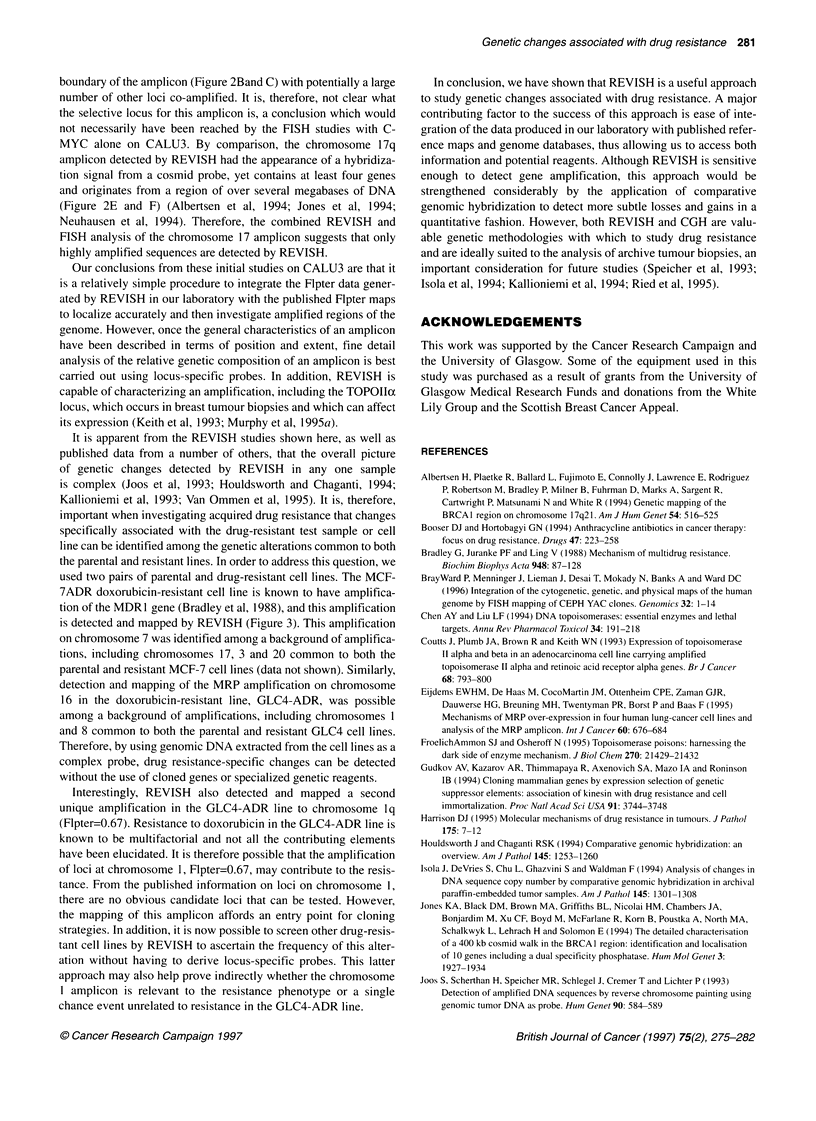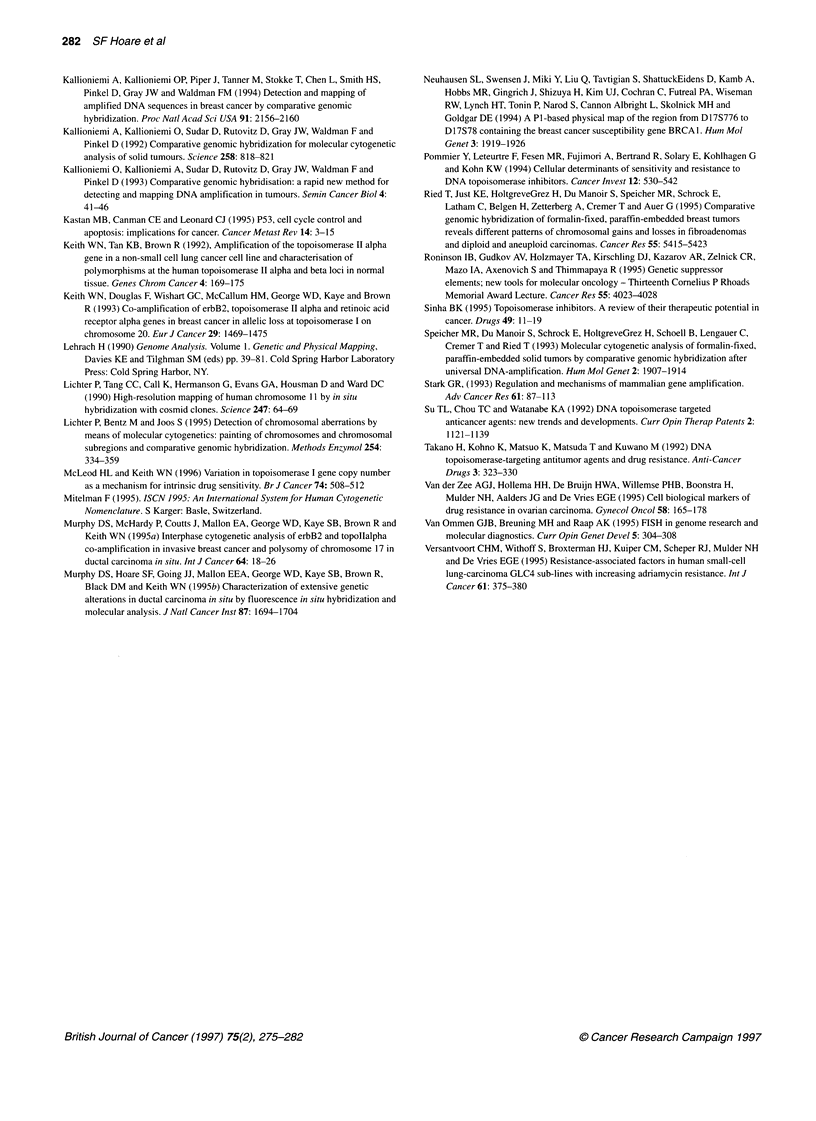# Identification of genetic changes associated with drug resistance by reverse in situ hybridization.

**DOI:** 10.1038/bjc.1997.45

**Published:** 1997

**Authors:** S. F. Hoare, C. A. Freeman, J. C. Coutts, J. M. Varley, L. James, W. N. Keith

**Affiliations:** CRC Department of Medical Oncology, University of Glasgow, CRC Beatson Laboratories, Bearsden, UK.

## Abstract

**Images:**


*British Journal of Cancer* (1997) **75**(2), 275–282
© 1997 Cancer Research Campaign

### Identification of genetic changes associated with drug resistance by reverse in situ hybridization

**SF Hoare[1], CA Freeman[1], JC Coutts[1], JM Varley[2], L James[2] and WN Keith[1]**

[1]CRC Department of Medical Oncology, University of Glasgow, CRC Beatson Laboratories, Alexander Stone Building, Garscube Estate, Switchback Road, Bearsden, Glasgow G61 1BD, UK; [2]CRC Department of Cancer Genetics, Paterson Institute for Cancer Research, Wilmslow Road, Manchester M20 9BX, UK

**Summary** The molecular cytogenetic techniques of comparative genomic hybridization (CGH) and reverse in situ hybridization (REVISH) allow the entire genomes of tumours to be screened for genetic changes without the requirement for specific probes or markers. In order to define the ability of REVISH to detect and map regions of amplification associated with drug resistance, we investigated a panel of cell lines selected for resistance to doxorubicin and intrinsic sensitivity to topoisomerase II-inhibitory drugs. We have defined a modified REVISH protocol, which involves double hybridizations with genomic DNA from the test cell lines and chromosome-specific whole chromosome paints to identify the chromosomes to which the amplicons localize. Sites of amplification are then mapped by fractional length measurements (Flpter), using published genome databases. Our findings show that amplification of the topoisomerase IIα gene is readily detected and mapped, as is amplification of the MDR and MRP loci. Interestingly, REVISH detected a new amplicon in the doxorubicin-resistant lung cancer cell line, GLC4-ADR, which mapped to chromosome 1q. REVISH is therefore ideally suited to characterize genetic changes specific for drug resistance within a background of genetic anomalies associated with tumour progression.

**Keywords:** molecular cytogenetics; fluorescence in situ hybridization; chromosome painting; drug resistance; gene amplification; gene mapping; reverse in situ hybridization

Many tumours respond to a range of cytotoxic agents. However, resistance often develops (van der Zee et al, 1995; Harrison, 1995). Understanding the mechanisms of resistance may provide new therapeutic options (Kastan et al, 1995; FroelichAmmon and Osheroff, 1995). At the cellular level, a number of resistance mechanisms can potentially operate. These mechanisms include drug efflux via membrane pumps, such as p-glycoprotein or multidrug resistance protein (MRP), drug metabolism, including inactivation or failure to activate a prodrug, an alteration in abundance of the target protein, for example topoisomerase II (topoII) enzyme, mutation of target protein and inactivation of pathways leading to cell death, such as apoptotic signalling (Booser and Hortobagyi, 1994; Harrison, 1995; Kastan et al, 1995; van der Zee et al, 1995). It is likely that in any one particular tumour, response to therapy is dependent on concurrent expression of multiple mechanisms. Even for extensively studied drugs, such as etoposide, which is a known topoII inhibitor, resistance is a complex issue (Su et al, 1992; Takano et al, 1992; Booser and Hortobagyi, 1994; Chen and Liu, 1994; Pommier et al, 1994; Sinha, 1995). The recent association of reduced kinesin expression with etoposide resistance, as identified by a genetic suppressor element approach, highlights the usefulness and requirement for new approaches to define potential components of the drug resistance repertoire (Gudkov et al, 1994; Roninson et al, 1995).

A major drawback to many of the conventional approaches used to investigate drug resistance mechanisms is that some prior information or guesswork on the changes that have occurred is required, thus necessitating separate reagents to screen each possible change. When analysing genetic changes for example, screening is limited to the use of gene- or region-specific probes. Recently, the molecular cytogenetic techniques of reverse in situ hybridization (REVISH) and its more advanced relative, comparative genomic hybridization (CGH), have been developed for the rapid global detection and mapping of genetic imbalances in tumour genomes (Kallioniemi et al, 1992, 1993; Joos et al, 1993; Houldsworth and Chaganti, 1994; Lichter et al, 1995; Mitelman, 1995; Van Ommen et al, 1995). In REVISH, genomic DNA from the tumour is used as a complex probe and hybridized to normal metaphase chromosomes (Mitelman, 1995). Genomic sequences amplified in the tumour are then detected as an increased intensity of signal at the normal chromosomal position from which the amplified sequences are derived (Joos et al, 1993; Lichter et al, 1995). For more accurate analysis of both loss and gain of genetic material, CGH is then required. However, CGH involves complex fluorescence ratioing techniques and expert knowledge of chromosome identification (Kallioniemi et al, 1993; Houldsworth and Chaganti, 1994; Lichter et al, 1995). However, both REVISH and CGH would seem to be ideal methods for detecting genetic changes associated with the acquisition of drug resistance in tumours.

In this study, we have developed a modified REVISH protocol, which can be applied in laboratories with minimal cytogenetic experience. This approach was used to investigate the genetics of drug sensitivity and resistance in a panel of human tumour cell lines.

### MATERIALS AND METHODS

### Cell lines

The cell lines used in this study (MCF-7, MCF-7ADR, GLC4, GLC4-ADR and CALU3) have been described previously

*Received 5 June 1996*
*Revised 15 August 1996*
*Accepted 20 August 1996*

*Correspondence to:* WN Keith

(Bradley et al, 1988; Coutts et al, 1993; Eijdems et al, 1995; Versantvoort et al, 1995).

### Extraction of genomic DNA

High molecular weight genomic DNA for REVISH was extracted from cell lines using a QIAGEN nucleic acid isolation kit (QIAGEN Ltd, Dorking, UK) according to manufacturer's protocols.

### Locus-specific DNA probes, chromosome paints and probe labelling

The C-MYC locus-specific probe and whole chromosome paint probes were purchased from Appligene Oncor (Durham, UK). ERBB-2 sequences were detected using a mixture of two cosmids, cRCNeul and cRCNeu4 (Murphy et al, 1995*a*). Topoisomerase II alpha (TOPOIIα) sequences were detected using cosmid ICRFC105b04155 (Murphy et al, 1995*b*), RARα sequences were detected using cosmid ICRFC105F1255, NF1 sequences were detected using cosmid ICRFC105c0861 and NM23 sequences were detected using cosmid ICRFC105H12160 developed from the Imperial Cancer Research Fund Reference Library (Lehrach, 1990). The VHR cosmid was obtained from Dr D Black, Beatson Institute, Glasgow. Commercial probes were ready labelled with digoxigenin (Murphy et al, 1995*b*). Cosmid probes were labelled with biotin as previously described (Murphy et al., 1995*a*). All hybridization conditions were regulated using the Omnislide modular in situ system (Hybaid, UK). Control hybridizations to lymphocytes allowed for the characterization of all the probes, which had hybridization efficiencies from 78% to 96% (McLeod and Keith, 1996), and for the determination of signal size and intensity required for quantitative analysis of gene copy number (Coutts et al, 1993).

### Labelling of genomic DNA for REVISH

Complete protocols for the labelling of genomic DNA are available from the authors on request (WNK, e-mail gpma59@udcf.gla.ac.uk). Briefly, 1.5 μg of genomic DNA was labelled with biotin and the fragment size range checked (500 bp–2 kb). The labelled probe was precipitated in the presence of 150 μg of cot-1 DNA and resuspended in 7 μl of hybridization mix. The probe was denatured and preannealed at 37°C for 1 h. For dual hybridizations with chromosome paints, the digoxigenin-labelled paint (7 μl) was also denatured and preannealed at 37°C for 2.5 h. The probe and paint were then mixed and added to denatured normal chromosomes and hybridized for 2–5 days. In order to control for the REVISH hybridizations, normal DNA extracted from lymphocytes was used in all experiments. The labelled normal DNA control reverse paints the chromosomes in an even fashion, with the expected exception of blocking of repetitive sequences (owing to inclusion of cot-1 DNA) at, for example, centromeric sequences. All hybridizations were carried out at least three times.

### Probe detection

Probe detection was described previously using the Hybaid Omnislide system (Murphy et al, 1995*a,b*). Fluorescence was analysed on a Bio-Rad (Richmond, CA, USA) MRC-600 laser scanning confocal microscope equipped with a krypton argon laser. Original unedited images were stored on optical disks and have been retained. All processed images were stored as separate files on optical disks. Optimal colour balance of the pseudocolour images was achieved using image-processing software (Photomagic, Micrografx, Arapaho Richardson, Texas, USA). Final figures were annotated in, and directly printed from, Micrografx Draw (Micrografx) using a dye sublimation printer (Colour Ease, Kodak, Harrow, UK).

### Fractional length measurements

Hybridization sites were localized by fractional length measurements (Flpter), in which the Flpter is the distance from the probe location to the end of the short arm of a chromosome divided by the total length of the chromosome (Lichter et al, 1990; McLeod and Keith, 1996). Analysis of digitized images for Flpter measurements was carried out using IPLab Spectrum software with SmartCapture extensions from Digital Scientific Ltd (Cambridge UK) (McLeod and Keith, 1996). In addition, GraphPolygon was used to produce an intensity plot of fluorescence along the profile of the chromosome where the width of the chromosome was determined in pixels and the average intensity over the width plotted (McLeod and Keith, 1996). Published Flpter maps are available from the Resource for Molecular Cytogenetics at Lawrence Berkeley National Laboratories and the University of California, San Francisco, USA (Internet connection, http://rmc-www.lbl.gov/), and also in BrayWard et al (1996). A minimum of five chromosomes were used to determine Flpter positions.

### Internet connections

A number of Internet connections were used to access genetic databases: (1) Resource for Molecular Cytogenetics at Lawrence Berkeley National Laboratories and the University of California, San Francisco, USA, Internet connection, http://rmc-www.lbl.gov/; (2) MRC, Human Genome Mapping Project (HGMP), Internet connection, http://www.hgmp.mrc.ac.uk/; (3) Online Mendelian Inheritance in Man (OMIM), Internet connection, http://www3.ncbi.nlm.nih.gov/Omim/.

### RESULTS

### Amplification of loci on chromosomes 8 and 17 in CALU3

In order to develop REVISH, we used the lung adenocarcinoma cell line CALU3 as a test system and subsequently as a control for REVISH, as we have considerable experience in using this cell line to study drug resistance (Coutts et al, 1993). Figure 1 shows examples of REVISH using test DNA extracted from CALU3 (Figure 1A and B) and normal DNA extracted from lymphocytes (Figure 1C and D) in combination with a chromosome 8 paint probe. The targets for hybridization are normal lymphocyte chromosomes. As can be seen from Figure 1C and D, the normal DNA control reverse paints the chromosomes in an even fashion, whereas DNA from CALU3 shows intensity changes associated with copy number alterations to sequences within the CALU3 genome. From the double hybridization of CALU3 DNA and chromosome 8 paint shown in Figure 1A and B, two regions of amplification are detected on chromosome 8.

The amplicon on the tip of the q-arm of chromosome 8 was

© Cancer Research Campaign 1997

**Figure 1** Reverse in situ hybridization of normal chromosomes with CALU3 DNA. (**A**) Image of normal human chromosomes after REVISH with biotin-labelled genomic DNA from CALU3 (detected with fluorescein isothiocyanate (FITC), pseudocoloured green) and a digoxigenin-labelled chromosome 8 paint (detected with Cy5, pseudocoloured red). Chromosomes are pseudocoloured blue. (**B**) Same hybridization as in **A** showing only the FITC image captured from the biotin-labelled genomic DNA from CALU3. This image shows the regional changes in sequence copy number more simply than the three-colour merged image in (**A**). (**C**) Image of normal human chromosomes after REVISH with biotin-labelled genomic DNA from normal lymphocytes (detected with FITC, pseudocoloured green) and a digoxigenin-labelled chromosome 8 paint (detected with Cy5, pseudocoloured red). Chromosomes are pseudocoloured blue. (**D**) Same hybridization as in **C** showing only the FITC image captured from the biotin-labelled genomic DNA from the lymphocytes. Chromosome 8 is marked in all images investigated in more detail to understand fully the principle of how this approach may be used to detect and map chromosomal amplifications. The two chromosome 8 amplicons were localized using fractional length measurements (Flpter) and assigned map positions of 0.29 and 0.82 (Figure 2A). Flpter maps for chromosome 8 published by the Resource for Molecular Cytogenetics at Lawrence Berkeley National Laboratories and the University of California, San Francisco, USA (Internet connection, http://rmc-

© Cancer Research Campaign 1997 

**Figure 2** Amplification of loci on chromosomes 8 and 17 in CALU3. **(A)** Detail of the FITC image from hybridization of biotin-labelled genomic DNA from CALU3 to normal chromosome 8. The two chromosome 8 amplicons at (1) Flpter=0.29 and (2) Flpter=0.82 are marked. **(B)** Detail of normal metaphase spread co-hybridized with genomic DNA from CALU3 (green) and a C-MYC locus-specific probe (red). Chromosomes are shown in blue. A visual representation of the hybridization site for C-MYC relative to the genomic sequences amplified in CALU3 is shown in **C**. **(C)** shows a typical intensity plot for fluorescence along the profile of chromosome 8 (blue trace), generating a visual representation of the hybridization site for C-MYC (red trace), relative to genomic sequences amplified in CALU3 (green trace). **(D)** Hybridization of C-MYC locus-specific probe (green) to metaphase spread chromosomes from the CALU3 cell line (red); showing amplification of C-MYC. **(E)** Detail of hybridization of the FITC image from biotin-labelled genomic DNA from CALU3 to normal chromosome 17. Both chromosome 17 homologues are shown and the amplicon has a Flpter of 0.49. **(F)** Detail of cosmid probes for chromosome 17 loci to chromosomes prepared from CALU3. Only the chromosome carrying the amplified loci is shown. Hybridization signal is shown in green, chromosomes in red. The loci for ERBB-2, TOPOIIα, RARα and VHR are amplified. **(G)** Structure of the chromosome 17 amplicon in CALU3 cells. Only the chromosome (blue) carrying the amplified loci is shown. (i) Double hybridization of chromosome 17 centromeric sequences (red) and TOPOIIα (green), showing linkage of the amplicon to chromosome 17 sequences. (ii) Double hybridization of chromosome 17 centromeric sequences (red) and NF1 (green), showing the position of NF1 close to the centromere as in normal chromosomes. (iii) Double hybridization of chromosome 17 centromeric sequences (red) and NM23H1 (green), showing the position of NM23H1 close to the centromere on the abnormal CALU3 chromosome 17. The normal locus order is centromere, NF1, ERBB-2, RARα, TOPOIIα, VHR and NM23H1. Thus, an inversion of chromosome 17 appears to have occurred in CALU3 to bring NM23H1 close to the centromere. (iv) Double hybridization of ERBB-2 (red) and TOPOIIα (green), showing the physical relationship between the co-amplified genes

 © Cancer Research Campaign 1997

**Figure 3** Reverse in situ hybridization of normal chromosomes with genomic DNA from drug-resistant cell lines. (**A**) Detail of REVISH hybridizations to normal chromosome 7 using the MCF-7 parental and resistant cell lines. Only the FITC images captured from the biotin-labelled genomic DNA from the lines is shown. (**B**) Detail of REVISH hybridizations to normal chromosome 16 using the GLC4 parental and resistant cell lines. Only the FITC images captured from the biotin-labelled genomic DNA from the lines is shown. (**C**) Detail of REVISH hybridizations to normal chromosome 1 using the GLC4 parental and resistant cell lines. Images of normal human chromosome 1 after REVISH hybridization with biotin-labelled genomic DNA from the GLC4 parental and resistant cell lines (detected with FITC, pseudocoloured green). Chromosomes are pseudocoloured red. The amplification at the telomeric region of chromosome 1q common to both the parental and resistant lines can be seen, as can the amplification unique to the resistant line. Thresholding was used to observe only the peak fluorescence intensities and these images are adjacent to the original images. The p-arm of each chromosome is marked with an arrow www.lbl.gov/), show the C-MYC oncogene to have a Flpter of 0.84 and it may therefore reside within the amplicon. This was analysed in two ways. Firstly, the physical relationship between the C-MYC locus and the amplicon was addressed by co-hybridizing a C-MYC probe with the genomic DNA from CALU3 in a modified REVISH experiment. As shown in Figure 2B and C, the C-MYC locus maps to the edge of the amplicon and may therefore define a marker close to the border of this rather extensive amplicon. Secondly, in order to test directly whether the C-MYC gene is amplified in CALU3, the C-MYC probe was hybridized to chromosomes prepared from CALU3 as shown in Figure 2D. Figure 2D shows that C-MYC is indeed amplified in CALU3 with copies distributed over a number of chromosomal sites, and so lies within the border of the amplicon (Figure 2C). However, it is worth noting that the amplicon extends over an area greater than just C-MYC (Figure 2B and 2C), and therefore numerous other loci are likely to be contained within this amplicon, a conclusion which would not have been reached had the cell line been analysed for C-MYC amplification by FISH alone. These experiments show the value and robust nature of using REVISH in combination with Flpter mapping and FISH to identify a previously uncharacterized amplicon and provide a strategy for analysis.

We have previously shown CALU3 to have co-amplification of the topoisomerase IIα (TOPOIIα) locus with other markers on chromosome 17, including ERBB-2 and RARα (Keith et al, 1992; Coutts et al, 1993), with the ERBB-2 locus amplified around 30-fold (Keith et al, 1992). We therefore compared the REVISH analysis with standard FISH to evaluate whether REVISH was capable of detecting the chromosome 17 amplicon in CALU3. Figure 2E shows detail of two chromosome 17 homologues after REVISH with CALU3 DNA, which clearly shows evidence of a site of amplification. The chromosome 17 amplicon was localized using Flpter and assigned a map position of 0.49. Flpter maps for chromosome 17 published by the Resource for Molecular Cytogenetics at Lawrence Berkeley National Laboratories and the University of California, San Francisco, USA, show the ERBB-2 oncogene to have a Flpter of 0.49, thus positioning the REVISH amplicon at a site consistent with our knowledge of gene amplification on chromosome 17 in CALU3 (Keith et al, 1992; Coutts et al, 1993).

However, in order to investigate further the relationship between the fluorescence signal generated by REVISH and the relative levels of amplification of specific loci within this region, metaphase preparations from the CALU3 cell line were analysed by FISH. Six cosmid probes localizing to chromosome 17q were used (NF1, ERBB-2, TOPOIIα, RARα, VHR and NM23H1) (Albertsen et al, 1994; Jones et al, 1994; Neuhausen et al, 1994). Figure 2F shows examples of hybridizations to a chromosome

© Cancer Research Campaign 1997 

carrying co-amplification of ERBB-2, TOPOIIα, RARα and VHR as a homogeneously staining region. The loci, NF1 and NM23H1, which reside either side of ERBB-2, TOPOIIα, RARα and VHR on chromosome 17q, are present as single copies and therefore define the limits of the amplicon. Interestingly, the amplified loci show variation in levels of amplification with ERBB-2 amplified to the greatest level followed by TOPOIIα. RARα and VHR are present as two small blocks of amplification (Figure 2F). Double hybridization experiments with these probes showed all the above loci to be still physically linked within the chromosome 17 carrying the amplicon (Figure 2G) and that there appears to be an inversion of the amplified region within chromosome 17 as defined by the hybridization position of the NM23H1 probe relative to the normal map positions of the chromosome 17 genes analysed. This pattern of amplification is consistent with unequal sister chromatid exchange mechanism of amplification (Stark 1993). Thus, this amplicon is derived from a region over 3 megabases in size, which on a normal chromosome encompasses the breast cancer susceptibility locus, BRCA1 (Albertsen et al, 1994; Jones et al, 1994; Neuhausen et al, 1994). The hybridization signal generated by REVISH shown in Figure 2E is therefore a product of a large amplicon originating from a region encompassing at least 3 megabases, yet the signal size and intensity are similar to that of a cosmid or P1 genomic probe used for standard FISH. This suggests that only the most highly amplified sequences are detected by the REVISH approach and that the true extent of the amplicon may only be detected by FISH using locus-specific cosmid probes.

### Amplification of loci in drug-resistant cell lines

A major objective of this study was to ascertain whether REVISH could detect chromosomal changes present between closely related pairs of cell lines selected for drug resistance from a sensitive parental line. Two paired lines were analysed, the breast line MCF-7 and its doxorubicin-resistant derivative, MCF-7ADR, and the small-cell lung cancer cell line GLC4 and its doxorubicin derivative, GLC4-ADR. REVISH analysis of the MCF-7 and MCF-7ADR lines identified a number of sites of amplification common to both lines and a site of amplification on chromosome 7q found only in the doxorubicin-resistant line. Figure 3 shows examples of the chromosome 7 REVISH signals for DNA extracted from MCF-7 and MCF-7ADR lines. The hybridization pattern for the parental line shows an imbalance in sequence between the p and q arms of chromosome 7, which is retained in the resistant line (Figure 3). However, the resistant line shows a very specific amplification mapping to 7q with a Flpter of 0.57. This localization is consistent with the map position of the multidrug resistance locus, MDR1, at 7q21.1, which is known to be amplified in the MCF-7 doxorubicin-resistant line and contributes to the drug-resistant phenotype of the line (Bradley et al, 1988).

A second doxorubicin-resistant cell line, GLC4-ADR, and its parental line were analysed by REVISH. As with the MCF-7 lines, REVISH identified a number of sites of amplification common to both the GLC4 and GLC4-ADR lines. However, two sites of amplification were present only in the GLC4-ADR line (Figure 3). The first amplicon mapped to chromosome 16p with a Flpter of 0.22. This localization is consistent with the map position of the locus coding for the multidrug resistance protein, MRP, at 16p13, which is known to be amplified in the GLC4 doxorubicin-resistant line and contributes to the drug-resistant phenotype of the line (Eijdems et al, 1995). Interestingly, a second amplicon mapped to chromosome 1q with a Flpter of 0.67. Figure 3 shows detail of the REVISH hybridization to chromosome 1 in both the GLC4 and the GLC4-ADR lines. Both lines have an amplicon mapping to the distal telomeric region of chromosome 1 with the amplicon specific to the resistant line present below the centromere and heterochromatic region of chromosome 1. The amplicon at Flpter = 0.67 in the resistant line has not been described previously and there is no clear candidate locus as yet mapped to this region that could participate in the resistance phenotype. Therefore, it is now important to identify the specific sequences present in this amplicon by a combination of approaches, including those described above, and test whether they do indeed contribute to the resistance phenotype.

### DISCUSSION

This study shows the application of reverse in situ hybridization to detect, map and characterize genetic changes associated with the sensitivity of cell lines to anti-cancer agents. In order to use REVISH for this purpose, a number of steps are carried out:

1 An initial reverse in situ hybridization is carried out using genomic DNA extracted from the test cell lines and compared with normal DNA controls (see Figure 1). A visual analysis of the resultant hybridization allows the key chromosomes with regional amplifications to be identified by their size and centromere position.
2 Confirmation of chromosomal identification is obtained by co-hybridizing the test cell line DNA with a whole chromosome paint and visualizing the two probes with different fluorochromes (see Figure 1). We have found this to be an important modification to standard REVISH protocols as it allows unambiguous chromosome identification in laboratories without cytogenetic experience.
3 Precise localization of the amplified sequences is carried out using fractional length measurements (Flpter).
4 Using the reference maps for Flpter measurements available through the Internet and also in BrayWard et al (1996), candidate loci or specific markers for the region of interest are identified.
5 Detailed genetic analysis of candidate loci are carried out by, for example, FISH (see Figure 2 for an example).
6 Using the information derived above, traditional positional cloning strategies can then be applied to the newly identified region of interest.

Initially, we applied the REVISH strategy to the CALU3 cell line as we have a considerable amount of knowledge about the genetic changes to the TOPOIIα locus in this line, and this therefore allowed us to determine how robust the above strategy for REVISH was (Coutts et al, 1993). Following the above schedule for REVISH analysis, two major sites of amplification were chosen for study in detail, the first on chromosome 8q and the second on chromosome 17q. By REVISH, the two amplicons had different appearances, with the chromosome 8q amplicon starting at a Flpter of 0.82 and extending to the distal tip of the chromosome, thus covering some 18% of the total length of the chromosome (Figures 1 and 2A, B and C). A particularly informative experiment was to co-localize the C-MYC probe to the chromosome 8 amplicon by co-hybridization experiments (Figure 2B and C). Thus, although the C-MYC gene is amplified in CALU3 (Figure 2D), it is at the

 © Cancer Research Campaign 1997

boundary of the amplicon (Figure 2Band C) with potentially a large number of other loci co-amplified. It is, therefore, not clear what the selective locus for this amplicon is, a conclusion which would not necessarily have been reached by the FISH studies with C-MYC alone on CALU3. By comparison, the chromosome 17q amplicon detected by REVISH had the appearance of a hybridization signal from a cosmid probe, yet contains at least four genes and originates from a region of over several megabases of DNA (Figure 2E and F) (Albertsen et al, 1994; Jones et al, 1994; Neuhausen et al, 1994). Therefore, the combined REVISH and FISH analysis of the chromosome 17 amplicon suggests that only highly amplified sequences are detected by REVISH.

Our conclusions from these initial studies on CALU3 are that it is a relatively simple procedure to integrate the Flpter data generated by REVISH in our laboratory with the published Flpter maps to localize accurately and then investigate amplified regions of the genome. However, once the general characteristics of an amplicon have been described in terms of position and extent, fine detail analysis of the relative genetic composition of an amplicon is best carried out using locus-specific probes. In addition, REVISH is capable of characterizing an amplification, including the TOPOIIα locus, which occurs in breast tumour biopsies and which can affect its expression (Keith et al, 1993; Murphy et al, 1995*a*).

It is apparent from the REVISH studies shown here, as well as published data from a number of others, that the overall picture of genetic changes detected by REVISH in any one sample is complex (Joos et al, 1993; Houldsworth and Chaganti, 1994; Kallioniemi et al, 1993; Van Ommen et al, 1995). It is, therefore, important when investigating acquired drug resistance that changes specifically associated with the drug-resistant test sample or cell line can be identified among the genetic alterations common to both the parental and resistant lines. In order to address this question, we used two pairs of parental and drug-resistant cell lines. The MCF-7ADR doxorubicin-resistant cell line is known to have amplification of the MDR1 gene (Bradley et al, 1988), and this amplification is detected and mapped by REVISH (Figure 3). This amplification on chromosome 7 was identified among a background of amplifications, including chromosomes 17, 3 and 20 common to both the parental and resistant MCF-7 cell lines (data not shown). Similarly, detection and mapping of the MRP amplification on chromosome 16 in the doxorubicin-resistant line, GLC4-ADR, was possible among a background of amplifications, including chromosomes 1 and 8 common to both the parental and resistant GLC4 cell lines. Therefore, by using genomic DNA extracted from the cell lines as a complex probe, drug resistance-specific changes can be detected without the use of cloned genes or specialized genetic reagents.

Interestingly, REVISH also detected and mapped a second unique amplification in the GLC4-ADR line to chromosome 1q (Flpter=0.67). Resistance to doxorubicin in the GLC4-ADR line is known to be multifactorial and not all the contributing elements have been elucidated. It is therefore possible that the amplification of loci at chromosome 1, Flpter=0.67, may contribute to the resistance. From the published information on loci on chromosome 1, there are no obvious candidate loci that can be tested. However, the mapping of this amplicon affords an entry point for cloning strategies. In addition, it is now possible to screen other drug-resistant cell lines by REVISH to ascertain the frequency of this alteration without having to derive locus-specific probes. This latter approach may also help prove indirectly whether the chromosome 1 amplicon is relevant to the resistance phenotype or a single chance event unrelated to resistance in the GLC4-ADR line.

In conclusion, we have shown that REVISH is a useful approach to study genetic changes associated with drug resistance. A major contributing factor to the success of this approach is ease of integration of the data produced in our laboratory with published reference maps and genome databases, thus allowing us to access both information and potential reagents. Although REVISH is sensitive enough to detect gene amplification, this approach would be strengthened considerably by the application of comparative genomic hybridization to detect more subtle losses and gains in a quantitative fashion. However, both REVISH and CGH are valuable genetic methodologies with which to study drug resistance and are ideally suited to the analysis of archive tumour biopsies, an important consideration for future studies (Speicher et al, 1993; Isola et al, 1994; Kallioniemi et al, 1994; Ried et al, 1995).

### ACKNOWLEDGEMENTS

This work was supported by the Cancer Research Campaign and the University of Glasgow. Some of the equipment used in this study was purchased as a result of grants from the University of Glasgow Medical Research Funds and donations from the White Lily Group and the Scottish Breast Cancer Appeal.

### REFERENCES

Albertsen H, Plaetke R, Ballard L, Fujimoto E, Connolly J, Lawrence E, Rodriguez P, Robertson M, Bradley P, Milner B, Fuhrman D, Marks A, Sargent R, Cartwright P, Matsunami N and White R (1994) Genetic mapping of the BRCA1 region on chromosome 17q21. *Am J Hum Genet* **54**: 516–525

Booser DJ and Hortobagyi GN (1994) Anthracycline antibiotics in cancer therapy: focus on drug resistance. *Drugs* **47**: 223–258

Bradley G, Juranke PF and Ling V (1988) Mechanism of multidrug resistance. *Biochim Biophys Acta* **948**: 87–128

BrayWard P, Menninger J, Lieman J, Desai T, Mokady N, Banks A and Ward DC (1996) Integration of the cytogenetic, genetic, and physical maps of the human genome by FISH mapping of CEPH YAC clones. *Genomics* **32**: 1–14

Chen AY and Liu LF (1994) DNA topoisomerases: essential enzymes and lethal targets. *Annu Rev Pharmacol Toxicol* **34**: 191–218

Coutts J, Plumb JA, Brown R and Keith WN (1993) Expression of topoisomerase II alpha and beta in an adenocarcinoma cell line carrying amplified topoisomerase II alpha and retinoic acid receptor alpha genes. *Br J Cancer* **68**: 793–800

Eijdems EWHM, De Haas M, CocoMartin JM, Ottenheim CPE, Zaman GJR, Dauwerse HG, Breuning MH, Twentyman PR, Borst P and Baas F (1995) Mechanisms of MRP over-expression in four human lung-cancer cell lines and analysis of the MRP amplicon. *Int J Cancer* **60**: 676–684

FroelichAmmon SJ and Osheroff N (1995) Topoisomerase poisons: harnessing the dark side of enzyme mechanism. *J Biol Chem* **270**: 21429–21432

Gudkov AV, Kazarov AR, Thimmapaya R, Axenovich SA, Mazo IA and Roninson IB (1994) Cloning mammalian genes by expression selection of genetic suppressor elements: association of kinesin with drug resistance and cell immortalization. *Proc Natl Acad Sci USA* **91**: 3744–3748

Harrison DJ (1995) Molecular mechanisms of drug resistance in tumours. *J Pathol* **175**: 7–12

Houldsworth J and Chaganti RSK (1994) Comparative genomic hybridization: an overview. *Am J Pathol* **145**: 1253–1260

Isola J, DeVries S, Chu L, Ghazvini S and Waldman F (1994) Analysis of changes in DNA sequence copy number by comparative genomic hybridization in archival paraffin-embedded tumor samples. *Am J Pathol* **145**: 1301–1308

Jones KA, Black DM, Brown MA, Griffiths BL, Nicolai HM, Chambers JA, Bonjardim M, Xu CF, Boyd M, McFarlane R, Korn B, Poustka A, North MA, Schalkwyk L, Lehrach H and Solomon E (1994) The detailed characterisation of a 400 kb cosmid walk in the BRCA1 region: identification and localisation of 10 genes including a dual specificity phosphatase. *Hum Mol Genet* **3**: 1927–1934

Joos S, Scherthan H, Speicher MR, Schlegel J, Cremer T and Lichter P (1993) Detection of amplified DNA sequences by reverse chromosome painting using genomic tumor DNA as probe. *Hum Genet* **90**: 584–589

Kallioniemi A, Kallioniemi OP, Piper J, Tanner M, Stokke T, Chen L, Smith HS, Pinkel D, Gray JW and Waldman FM (1994) Detection and mapping of amplified DNA sequences in breast cancer by comparative genomic hybridization. *Proc Natl Acad Sci USA* **91**: 2156–2160

Kallioniemi A, Kallioniemi O, Sudar D, Rutovitz D, Gray JW, Waldman F and Pinkel D (1992) Comparative genomic hybridization for molecular cytogenetic analysis of solid tumours. *Science* **258**: 818–821

Kallioniemi O, Kallioniemi A, Sudar D, Rutovitz D, Gray JW, Waldman F and Pinkel D (1993) Comparative genomic hybridisation: a rapid new method for detecting and mapping DNA amplification in tumours. *Semin Cancer Biol* **4**: 41–46

Kastan MB, Canman CE and Leonard CJ (1995) P53, cell cycle control and apoptosis: implications for cancer. *Cancer Metast Rev* **14**: 3–15

Keith WN, Tan KB, Brown R (1992), Amplification of the topoisomerase II alpha gene in a non-small cell lung cancer cell line and characterisation of polymorphisms at the human topoisomerase II alpha and beta loci in normal tissue. *Genes Chrom Cancer* **4**: 169–175

Keith WN, Douglas F, Wishart GC, McCallum HM, George WD, Kaye and Brown R (1993) Co-amplification of erbB2, topoisomerase II alpha and retinoic acid receptor alpha genes in breast cancer in allelic loss at topoisomerase I on chromosome 20. *Eur J Cancer* **29**: 1469–1475

Lehrach H (1990) *Genome Analysis.* Volume 1. *Genetic and Physical Mapping*, Davies KE and Tilghman SM (eds) pp. 39–81. Cold Spring Harbor Laboratory Press: Cold Spring Harbor, NY.

Lichter P, Tang CC, Call K, Hermanson G, Evans GA, Housman D and Ward DC (1990) High-resolution mapping of human chromosome 11 by *in situ* hybridization with cosmid clones. *Science* **247**: 64–69

Lichter P, Bentz M and Joos S (1995) Detection of chromosomal aberrations by means of molecular cytogenetics: painting of chromosomes and chromosomal subregions and comparative genomic hybridization. *Methods Enzymol* **254**: 334–359

McLeod HL and Keith WN (1996) Variation in topoisomerase I gene copy number as a mechanism for intrinsic drug sensitivity. *Br J Cancer* **74:** 508–512

Mitelman F (1995). *ISCN 1995: An International System for Human Cytogenetic Nomenclature.* S Karger: Basle, Switzerland.

Murphy DS, McHardy P, Coutts J, Mallon EA, George WD, Kaye SB, Brown R and Keith WN (1995*a*) Interphase cytogenetic analysis of erbB2 and topoIIalpha co-amplification in invasive breast cancer and polysomy of chromosome 17 in ductal carcinoma *in situ*. *Int J Cancer* **64**: 18–26

Murphy DS, Hoare SF, Going JJ, Mallon EEA, George WD, Kaye SB, Brown R, Black DM and Keith WN (1995*b*) Characterization of extensive genetic alterations in ductal carcinoma *in situ* by fluorescence *in situ* hybridization and molecular analysis. *J Natl Cancer Inst* **87**: 1694–1704

Neuhausen SL, Swensen J, Miki Y, Liu Q, Tavtigian S, ShattuckEidens D, Kamb A, Hobbs MR, Gingrich J, Shizuya H, Kim UJ, Cochran C, Futreal PA, Wiseman RW, Lynch HT, Tonin P, Narod S, Cannon Albright L, Skolnick MH and Goldgar DE (1994) A P1-based physical map of the region from D17S776 to D17S78 containing the breast cancer susceptibility gene BRCA1. *Hum Mol Genet* **3**: 1919–1926

Pommier Y, Leteurtre F, Fesen MR, Fujimori A, Bertrand R, Solary E, Kohlhagen G and Kohn KW (1994) Cellular determinants of sensitivity and resistance to DNA topoisomerase inhibitors. *Cancer Invest* **12**: 530–542

Ried T, Just KE, HoltgreveGrez H, Du Manoir S, Speicher MR, Schrock E, Latham C, Belgen H, Zetterberg A, Cremer T and Auer G (1995) Comparative genomic hybridization of formalin-fixed, paraffin-embedded breast tumors reveals different patterns of chromosomal gains and losses in fibroadenomas and diploid and aneuploid carcinomas. *Cancer Res* **55**: 5415–5423

Roninson IB, Gudkov AV, Holzmayer TA, Kirschling DJ, Kazarov AR, Zelnick CR, Mazo IA, Axenovich S and Thimmapaya R (1995) Genetic suppressor elements; new tools for molecular oncology – Thirteenth Cornelius P Rhoads Memorial Award Lecture. *Cancer Res* **55**: 4023–4028

Sinha BK (1995) Topoisomerase inhibitors. A review of their therapeutic potential in cancer. *Drugs* **49**: 11–19

Speicher MR, Du Manoir S, Schrock E, HoltgreveGrez H, Schoell B, Lengauer C, Cremer T and Ried T (1993) Molecular cytogenetic analysis of formalin-fixed, paraffin-embedded solid tumors by comparative genomic hybridization after universal DNA-amplification. *Hum Mol Genet* **2**: 1907–1914

Stark GR, (1993) Regulation and mechanisms of mammalian gene amplification. *Adv Cancer Res* **61**: 87–113

Su TL, Chou TC and Watanabe KA (1992) DNA topoisomerase targeted anticancer agents: new trends and developments. *Curr Opin Therap Patents* **2**: 1121–1139

Takano H, Kohno K, Matsuo K, Matsuda T and Kuwano M (1992) DNA topoisomerase-targeting antitumor agents and drug resistance. *Anti-Cancer Drugs* **3**: 323–330

Van der Zee AGJ, Hollema HH, De Bruijn HWA, Willemse PHB, Boonstra H, Mulder NH, Aalders JG and De Vries EGE (1995) Cell biological markers of drug resistance in ovarian carcinoma. *Gynecol Oncol* **58**: 165–178

Van Ommen GJB, Breuning MH and Raap AK (1995) FISH in genome research and molecular diagnostics. *Curr Opin Genet Devel* **5**: 304–308

Versantvoort CHM, Withoff S, Broxterman HJ, Kuiper CM, Scheper RJ, Mulder NH and De Vries EGE (1995) Resistance-associated factors in human small-cell lung-carcinoma GLC4 sub-lines with increasing adriamycin resistance. *Int J Cancer* **61**: 375–380

© Cancer Research Campaign 1997